# Cardiometabolic risk factors correlated with the incidence of dysglycaemia in a Brazilian normoglycaemic sample: the Baependi Heart Study cohort

**DOI:** 10.1186/s13098-019-0512-0

**Published:** 2020-01-13

**Authors:** Camila Maciel De Oliveira, Luciane Viater Tureck, Danilo Alvares, Chunyu Liu, Andrea Roseli Vançan Russo Horimoto, Rafael de Oliveira Alvim, José Eduardo Krieger, Alexandre C. Pereira

**Affiliations:** 1 Laboratory of Genetics and Molecular Cardiology, Heart Institute (InCor), University of São Paulo Medical School, Sao Paulo, Brazil; 2 Department of Integrative Medicine, Federal University of Parana, Curitiba, Brazil; 3 Global Co-creation Lab, Institute for Medical Engineering and Science, Massachussets Institute of Tecnology (MIT), Cambridge, USA; 4 Department of Genetics, Federal University of Parana, Curitiba, Brazil; 5 Department of Statistics, Pontifícia Universidad Católica de Chile, Santiago, Chile; 6 Framingham Heart Study, Framingham, USA; 7 Department of Biostatistics, Boston University, Boston, USA; 8 Department of Physiological Sciences, Federal University of Amazonas, Manaus, Brazil

**Keywords:** Baependi Heart Study cohort, Dysglycaemia, Cardiovascular risk factors, Brazil, Longitudinal study, Cohort

## Abstract

**Background:**

Dysglycaemia is defined by elevated glucose levels in the blood, commonly characterized by impaired fasting glucose, impaired glucose tolerance, elevated glycated haemoglobin, or diabetes mellitus (DM) diagnosis. The abnormal levels of glucose may occur many years before DM, a condition known as prediabetes, which is correlated with comorbidities such as cardiovascular diseases. Therefore, the aim of this study was to investigate the incidence of prediabetic dysglycaemia and its relationship with cardiometabolic risk factors at a 5-year follow-up, based on an initially normoglycaemic sample in the Baependi Heart Study cohort.

**Methods:**

The data used comes from the Baependi Heart Study cohort, which consists of two periods: cycle 1 (2005–2006) and cycle 2 (2010–2013). For this study, we excluded those who had fasting blood glucose ≥ 100 mg/dL or were taking anti-diabetic medications at baseline, and those that had diabetes diagnosed in cycle 2. Mixed-effects logistic regression models were used to assess the association between cardiometabolic risk factors and the incidence of dysglycaemia, including a familiar random effect such as a cluster.

**Results:**

The incidence of prediabetic dysglycaemia was 12.8%, and it did not differ between men and women (14.4% and 11.6%, respectively). Two models were analysed to investigate the relationship between cardiometabolic risk factors and the occurrence of prediabetic dysglycaemia. The model that better explained the occurrence of dysglycaemia over the 5 years, after correction, included the waist circumference (WC) (measures and Δ), systolic blood pressure (SBP), HDL-c levels, and age. Although sex was not associated with the incidence of dysglycaemia, women and men showed differences in cardiometabolic risk factors related to glucose impairment: men who developed dysglycaemia showed, in parallel, higher LDL-c levels, TC/HDL-c ratio and DBP measurements; while these parameters remained similar between women who developed dysglycaemia and dysglycaemia-free women, after 5 years.

**Conclusions:**

In an initially normoglycaemic sample of a highly mixed population living in a traditional Brazilian lifestyle, important cardiometabolic risk factors were associated with the occurrence of prediabetic dysglycaemia, and this relationship appeared to be more important in men. These results provide important insights about cardiovascular risk in prediabetic individuals.

## Background

The term dysglycaemia means, broadly, the abnormality of glucose levels. The glucose impairment can occur many years before type 2 diabetes mellitus (T2DM), establishing a condition known as prediabetes. Even though this metabolic disorder is initial, the risk to the development of some comorbidities, such as cardiovascular disease, seems to increase [1]. Identifying individuals at the earliest stage of dysglycaemia would be useful to allow the adoption of strategies for preventing or delaying the progression of the disease.

The prediabetes stage is identified by impaired fasting blood glucose (IFG), impaired glucose tolerance (IGT), or impaired glucose regulation [glycated haemoglobin—HbA1c between 42 and 47 mmol/mol (6.0–6.4%)] [2], and it indicates the risk of developing T2DM.

Although fasting blood glucose has some limitations as a population screening technique, it is the most used test for dysglycaemia, particularly in developing countries. The identification of correlated variables has a particular importance in the knowledge about underlying etiologies of dysglycaemia and novel biomarkers that could provide greater discrimination of future risk. In this context, novel genetic and clinical scores have also been proposed [3–5].

The relationship between prediabetic dysglycaemia and other associated risk factors has been studied across the world [1, 6, 7], but there is no consensus, since each population has particularities regarding environmental, genetic and clinical factors involved. The identification of the most important conditions related to the incidence of prediabetic dysglycaemia is needed to allow the development of specific strategies to prevent the modifiable risk factors. Thus, the aim of this study was to investigate which cardiometabolic risk factors, or their variations over time, could be correlated with the incidence of prediabetic dysglycaemia, at a 5-year follow-up in a Brazilian normoglycaemic sample.

## Methods

### Study population

This study is part of a larger project: The Baependi Heart Study, that has a longitudinal design seeking to observe cardiovascular risk factors [8] and other prevalent non-communicable chronic diseases, including patients of both sexes aged 18 years or above. For this study, we carried out a cross-sectional analysis of the data collected in two distinct periods: baseline (cycle 1, 2005–2006) [8] and 5-year follow-up (cycle 2, 2010–2013) [9].

At the baseline, 95 families (1695 individuals) were selected in Baependi, Minas Gerais State, Brazil (752 km^2^, 19,117 inhabitants). At the 5-year follow-up, 2495 individuals distributed in 125 families were evaluated. Probands were identified from the community-at-large in several stages and included all living relatives in the town [8]. For clinical exams and physical examination, a clinic was established in the center of the city.

Each subject provided informed written consent that was approved by the ethics committee of the Hospital das Clínicas (SDC: 3485/10/074), University of São Paulo, Brazil.

### Samples

We excluded those participants who had no matched data—baseline and 5-year (n = 608). We also excluded participants who had fasting blood glucose (FBG) ≥ 100 mg/dL or were taking anti-diabetic medications (n = 249) at baseline. To determine the incidence of prediabetic dysglycaemia, we further excluded participants who had developed diabetes at the 5-year follow-up (n = 56). The final participants (n = 799) remained for statistical analysis.

### Clinical characteristics

A protocol was defined for investigating general and medical information. Waist circumference (WC), hip circumference (HC), body mass index (BMI), systolic (SBP) and diastolic blood pressure (DBP) were measured according to established procedures. FBG, triglycerides (TG), total cholesterol (TC) and high density lipoprotein cholesterol (HDL-c) were evaluated by standard techniques in 12-h fasting blood samples. We also analyzed the ratio of waist circumference to hip circumference (WHR) and TC to HDL-c levels (total/HDL-c ratio). Dysglycaemia was defined as FBG ≥ 100 mg/dL and < 126 mg/dL. T2DM was defined as FBG ≥ 126 mg/dL or the use of hypoglycemic medications.

### Statistical analysis

Descriptive analyses were shown as mean ± SD for continuous and percentage for categorical variables. The comparisons of categorical covariates were performed by the Chi square test, and the means were compared by the Student’s t-test. The differences (Δ values) were estimated by subtracting the cycle 2 value from the cycle 1 value.

The analysis focused on the search for the best model for assessing the incidence of dysglycaemia in this initially normoglycaemic sample. Because all individuals were followed-up at 5 years, we used mixed effect logistic regression models (accounting for relatedness) to assess the association between clinical covariates and a dysglycaemia diagnosis. For model 1, independent variables included age, sex, SBP, TG, HDL-c, BMI, FBG and Δ values. For model 2, BMI and ΔBMI were substituted with WC and ΔWC. These variables were chosen based on biological plausibility. To evaluate the performance of the proposed models, receiver operating characteristic (ROC) curves were built and the Akaike information criterion (AIC) was used to measure the discriminatory power for dysglycaemia using the two models. All continuous covariates were log transformed and standardized before modeling. Statistical analyses were carried out using R software (version 3.5.1) and the level of significance was set at p < 0.05.

## Results

In the normoglycaemic sample, both men and women had a similar mean age (42 ± 17 years for men and 40 ± 15 years for women) at baseline and presented a similar set of characteristics changing over the 5 years, but SBP and DBP changed only among women (Table 1). Anthropometric variables, FBG, TC, LDL-c, CT-c/HDL-c increased, and HDL-c decreased for both men and women (p < 0.05), while TG did not alter at the 5-year follow-up (Table 1).

**Table 1:** Clinical characteristics by sex in the Baependi Heart Study

Variables	Men			Women		
	Baseline	5-year follow-up	*p*-valu*e*	Baseline	5-year follow-up	*p*-value
SBP (mmHg)	128.73 ± 18.0	128.32 ± 16.9	0.750	120.52 ± 17.3	123.30 ± 15.9	*0.010*
DBP (mmHg)	78.24 ± 11.3	77.47 ± 10.9	0.358	77.4 ± 10.7	76.08 ± 10.1	*0.046*
BMI (kg/m^2^)	23.16 ± 3.7	25.34 ± 4.6	< *0.001*	25.01 ± 5.1	26.16 ± 5.3	< *0.001*
WC (cm)	85.38 ± 10.9	91.01 ± 12.6	< *0.001*	86.55 ± 12.2	91.59 ± 12.0	< *0.001*
WHR	0.90 ± 0.08	0.94 ± 0.09	< *0.001*	0.87 ± 0.08	0.92 ± 0.07	< *0.001*
FBG (mg/dL)	81.27 ± 11.4	94.13 ± 20.9	< *0.001*	79.85 ± 12.4	92.20 ± 21.3	< *0.001*
TC (mg/dL)	172.85 ± 47.2	201.09 ± 42.0	< *0.001*	177.63 ± 47.1	202.54 ± 89.0	< *0.001*
HDL-c (mg/dL)	53.76 ± 14.9	45.49 ± 10.7	< *0.001*	57.49 ± 15.7	48.79 ± 12.1	< *0.001*
TC/HDL-c ratio	3.47 ± 1.56	4.61 ± 1.35	< *0.001*	3.32 ± 1.31	4.36 ± 2.18	< *0.001*
LDL-c (mg/dL)	92.78 ± 42.2	127.11 ± 35.9	< *0.001*	95.8 ± 43.6	124.17 ± 35.1	< *0.001*
TG (mg/dL)	132.5 ± 80.2	141.76 ± 88.5	0.137	125.10 ± 63.1	128.24 ± 66.9	0.448

Results are expressed as mean ± standard deviation; comparisons are between baseline and 5-year follow-up in each sex

Statistically significant *p* values are in italic (*p* < 0.05)

*SBP* systolic blood pressure, *DBP* diastolic blood pressure, *BMI* body mass index, *WC* waist circumference, *WHR* waist hip ratio, *FBG* fasting blood glucose, *TC* total cholesterol, *HDL-c* high density lipoprotein cholesterol, *TC/HDL-c* total cholesterol:HDL-c ratio, *LDL-c* low density lipoprotein cholesterol; *TG* triglycerides

The incidence of prediabetic dysglycaemia was 12.8% at the 5-year follow-up in this initially normoglycaemic sample, and it did not differ between men (14.4%) and women (11.6%). Women who developed dysglycaemia were older and presented higher SBP, DBP, and WHR at baseline when compared to the dysglycaemia-free group (Table 2), while only BMI was different among the men. After 5 years, among those with dysglycaemia, worse clinical and laboratory characteristics were observed (Table 2). For women, BMI, WC, and TG also became worse among those in the dysglycaemic group over time, while men showed significant changes for all variables, except HDL-c, in a period of 5 years. However, DBP was similar between women with and without dysglycaemia at the 5-year follow-up.

**Table 2:** Clinical characteristics by sex and glycaemic status in the Baependi Heart Study

	Baseline			5-year follow-up		
	Remained dysglycaemia-free	Developed dysglycaemia	*p*-value	Remained dysglycaemia-free	Developed dysglycaemia	*p*-value
Men						
Age (years)	41.52 ± 17.0	44.96 ± 16.0	0.173	46.17 ± 15.9	51.57 ± 14.0	*0.022*
SBP (mmHg)	128.28 ± 18.0	130.47 ± 18.8	0.449	126.56 ± 16.6	133.81 ± 16.0	*0.005*
DBP (mmHg)	77.84 ± 11.3	80.62 ± 12.5	0.150	76.48 ± 10.6	82.91 ± 11.8	*0.0009*
BMI (kg/m^2^)	22.98 ± 3.6	24.77 ± 4.8	*0.017*	24.64 ± 4.3	28.20 ± 5.0	< *0.001*
WC (cm)	85 ± 11	88.16 ± 11.9	0.082	89.19 ± 12.2	97.57 ± 11.6	< *0.001*
WHR	0.90 ± 0.08	0.92 ± 0.10	0.194	0.93 ± 0.09	0.97 ± 0.07	*0.0006*
FBG (mg/dL)	80.80 ± 11.4	83.29 ± 11.8	0.175	87.53 ± 7.6	107.53 ± 6.1	<*0.001*
TC (mg/dL)	173.74 ± 49.0	175.50 ± 45.1	0.803	197.16 ± 39.5	221.06 ± 46.7	*0.001*
HDL-c (mg/dL)	54.32 ± 14.2	54.50 ± 19.8	0.952	45.74 ± 10.4	44.84 ± 12.3	0.629
TC/HDL-c ratio	3.43 ± 1.57	3.65 ± 1.74	0.402	4.50 ± 1.28	5.18 ± 1.51	*0.003*
LDL-c (mg/dL)	93.21 ± 43.3	92.02 ± 43.9	0.860	125.73 ± 34.2	139.86 ± 40.9	*0.028*
TG (mg/dL)	132.42 ± 82.0	144.63 ± 75.7	0.305	128.02 ± 70.7	184.14 ± 116.9	*0.001*
Women						
Age (years)	38.82 ± 14.5	47.08 ± 14.2	*0.0002*	43.47 ± 14.0	51.87 ± 14.3	*0.0003*
SBP (mmHg)	119.37 ± 16.6	126.76 ± 19.0	*0.008*	122.08 ± 15.5	128.12 ± 15.9	*0.012*
DBP (mmHg)	76.92 ± 10.5	80.70 ± 10.7	*0.018*	75.83 ± 10.1	77.46 ± 9.5	0.252
BMI (kg/m^2^)	24.89 ± 5.1	25.13 ± 4.3	0.716	25.50 ± 4.9	28.98 ± 5.2	< *0.001*
WC (cm)	85.97 ± 12.0	88.98 ± 10.7	0.062	90.14 ± 12.0	96.52 ± 10.7	*0.0002*
WHR	0.87 ± 0.08	0.89 ± 0.07	*0.045*	0.91 ± 0.07	0.95 ± 0.07	*0.0007*
FBG (mg/dL)	79.62 ± 12.3	84.32 ± 11.7	*0.008*	86.46 ± 7.7	107.57 ± 7.4	<* 0.001*
TC (mg/dL)	176.68 ± 46.6	186.80 ± 51.6	0.179	201.22 ± 95.2	206.32 ± 37.7	0.468
HDL-c (mg/dL)	57.26 ± 15.8	58.61 ± 14.6	0.534	49.20 ± 12.0	47.34 ± 13.2	0.336
TC/HDL-c ratio	3.32 ± 1.33	3.35 ± 1.15	0.891	4.27 ± 2.24	4.69 ± 1.62	0.101
LDL-c (mg/dL)	95.04 ± 42.0	103.12 ± 56.41	0.318	123.23 ± 33.9	129.27 ± 35.1	0.246
TG (mg/dL)	124.67 ± 64.3	134.79 ± 57.2	0.237	121.46 ± 60.6	147.02 ± 63.0	*0.006*

Mean ± standard deviation

Statistically significant *p* values are in italic (*p* < 0.05)

*SBP* systolic blood pressure, *DBP* diastolic blood pressure, *BMI* body mass index, *WC* waist circumference, *WHR* waist hip ratio, *FBG* fasting blood glucose, *TC* total cholesterol, *HDL-c* high density lipoprotein cholesterol, *TC/HDL-c* total cholesterol: HDL-c ratio, *LDL-c* low density lipoprotein cholesterol, *TG* triglycerides

We also identified the best model to predict the incidence of dysglycaemia in a normoglycaemic population using the set of evaluated characteristics. Two models were selected. The models differ regarding the presence of BMI and ΔBMI (model 1), or the presence of WC and ΔWC (model 2) (Table 3). Based on the ROC and AIC values, model 2 presented the better combination of variables that distinguished individuals who developed dysglycaemia from those who remained dysglycaemia-free over the 5 years (model 1: ROC: 0.870, AIC: 428.474; model 2: ROC: 0.857, AIC: 447.588). In both models, age, ΔFBG, SBP, and HDL-c were associated with the incidence of dysglycaemia (Table 3). When including BMI and ΔBMI into the model (model 1), both were significant. If including WC and ΔWC (model 2), both were related to the incidence of dysglycaemia as well.

**Table 3:** Cross-sectional correlates of clinical characteristics at baseline and dysglycaemia status at 5-year follow-up in the Baependi Heart Study

Covariates	Model 1		Model 2	
	β (SD)	*p*-value	β (SD)	*p*-value
Age	0.42 (0.16)	*0.0068*	0.39 (0.15)	*0.0113*
Sex	0.06 (0.29)	0.8262	0.09 (0.27)	0.7376
ΔFBG	2.00 (0.25)	< *0.001*	2.02 (0.24)	< *0.001*
SBP	0.33 (0.19)	0.0887	0.37 (0.18)	*0.0426*
TG	0.38 (0.18)	0.3909	0.33 (0.17)	0.1996
HDL-c	0.04 (0.17)	*0.0260*	0.04 (0.17)	*0.0433*
BMI	0.23 (0.15)	*0.0022*		
WC			0.28 (0.15)	*0.0021*
ΔSBP	0.16 (0.21)	0.8668	0.22 (0.20)	0.8546
ΔTG	0.47 (0.21)	0.5276	0.46 (0.20)	0.4194
ΔHDL-c	0.13 (0.17)	0.1871	0.16 (0.17)	0.0912
ΔBMI	0.63 (0.17)	*0.0001*		
ΔWC			0.45 (0.16)	*0.0042*

Model 1, accounting for body mass index and Δ body mass index; Model 2, accounting for waist circumference and Δ waist circumference

Statistically significant *p* values are in italic (*p* < 0.05)

*β* regression coefficient, *SE* standard error of β, *FBG* fasting blood glucose, *SBP* systolic blood pressure, *TG* triglycerides, *HDL-c* high density lipoprotein cholesterol, *BMI* body mass index, *WC* waist circumference

## Discussion

In our study we observed that individuals showed a worsening of the cardiometabolic risk factors in parallel to prediabetic dysglycaemia development, in both sexes, compared to individuals that remained dysglycaemia-free over the 5 years. However, in men, the relationship between prediabetic dysglycaemia and the worsening of the cardiovascular factors may be even closer, since the LDL-c levels, TC/HDL-c ratio and DBP measurements were higher in men that developed dysglycaemia. These parameters remained similar between women with and without dysglycaemia at 5 years.

In this sense, at baseline, women showed more differences between who developed dysglycaemia over the 5 years and who remained dysglycaemia-free; while in men only BMI was initially different (at baseline). This may indicate that in men the metabolic impairment seemed to advance faster, compared to women in the same period and age range.

Taken together, these observations highlight sex-differences that may have implications for the follow-up and treatment of prediabetic individuals. However, the mechanisms underlying these sex-differences are not fully understood. What is known is that sex hormones have a significant influence on glucose and other metabolic parameters.

The reduced levels of ovarian hormones in postmenopausal women lead to an altered body fat distribution, mainly the visceral fat, and an increased incidence of diabetes and other cardiovascular risk factors [10, 11]. With age, increased body fat and risk of cardiovascular disease may be observed among men because of lower testosterone levels [12]. Underlying these inverse correlations, we have complex interactions between these hormones and adipose tissue distribution, production of cytokines and adipokines, hepatic gluconeogenesis, glucose uptake by skeletal muscle, genetic factors and the gut microbiome, differently affecting glucose regulation in men and women [10].

It is also important consider that these finds are related to a particular population: a lower-income population with traditionally rural habits. In other populations, with different characteristics, these sex-differences may not be observed. Therefore, it is important to conduct these studies in different populations, since our study until now is the first to analyze this relationship.

Regardless of sex, the prediabetic dysglycaemia is the intermediate metabolic stage between normal glucose and diabetes mellitus (DM) [13], thus, it is an important clinical alert to the risk for developing DM: annually, 5–10% of prediabetic individuals progress to T2DM and a similar proportion return to normoglycaemia [14].

In general, prediabetes has increased around world [15]. Based on impaired glucose tolerance (IGT), a study estimated an increase of prediabetes from 15.4% (2017) to 16.7% (2045) in North America and Caribbean [15]. The same study estimated an increase of prediabetes from 10% (2017) to 11.5% (2045) in South and Central America [15]. But, it is important to consider that estimations of prediabetes prevalence and incidence can differ widely, because there is no consensus in the screening criteria and definitions [15]. In Brazil, there are few studies that estimated the prediabetes prevalence in a large sample, in this sense, one of the main studies from Brazilian population investigated 15,105 civil servants aged 35–74 years (2008–2010), and found intermediate hiperglycaemia ranged from 16.1 to 52.6%, following various criteria [16]. Compared to our study, the incidence of prediabetes was higher, since we found 12.8%. It can be due the screening criteria and also due the population habits, since the previous study counted with an urban sample.

In addition to the risk of progressing to diabetes, the prediabetes itself is a serious health condition. Several studies suggest that individuals with prediabetes, already present metabolic alterations related to cardiovascular risk. The Diabetes Insulin Glucose and Myocardial Infarction (DIGAMI) study, investigating at two times (a few days after a myocardial infarction (MI), then 3 months later), found a 35% prevalence of dysglycaemia in patients with an acute (MI) [17]. The relationship between glycaemia and cardiovascular risk (cardiovascular disease, coronary heart disease, and stroke) seems to start when the glucose levels are normal [13, 18]. Jung and collaborators showed that the worsening of the glycaemic status was a determinant factor for the increased risk of hypertension [19]. In a meta-analysis with a large sample, the prediabetes defined by impaired fasting glucose or impaired glucose tolerance, was associated with a set of cardiovascular events, even in people with fasting glucose concentration as low as 5.55 mmol/L [1].

In this sense, our results showed that—when corrected for sex, age and ΔFBG—the systolic blood pressure, HDL-c levels and increased BMI were predictors of dysglycaemia over the 5 years in this population. This finding correlates again with the relationship between impaired glucose metabolism and other risk factors for cardiovascular diseases. Similar findings have provided a basis for the “ticking-clock” hypothesis [20]. In the San Antonio Heart Study (SAHS), the development of type 2 diabetes over 8 years of follow-up was accompanied by increased LDL-c and TG levels, BMI and blood pressure, very similar to what we observed in our study [20]. According to the “ticking-clock” hypothesis, the clock for cardiovascular disease begins to run before the T2DM diagnosis, since microvascular complications are established by the time T2DM is diagnosed [20].

Our study had some important limitations. Confounding variables, such as physical activity levels and dietary patterns, may be important factors to consider in some of our analyses. Besides, it will provide important information in the context of this study. The assessment of dysglycaemia by IGT and/or IFG may provide more accurate data regarding the prediabetic status; however, this is not feasible in many circumstances. However, to the best of our knowledge, this is the first study to investigate the incidence of prediabetic dysglycaemia and its relationship with cardiometabolic factors risk in a small rural population from Brazil.

## Conclusions

In conclusion, the incidence of prediabetic dysglycaemia in the Baependi population was accompanied by worsening of the cardiovascular risk factors in both men and women. However, there was heterogeneity between the sexes regarding the set of characteristics related to dysglycaemia and its progression. These results emphasize the importance of conducting studies in specific populations to understand this relationship in more detail, and also to promote screening for cardiovascular risk in prediabetic individuals. It is also important emphasize the role of lifestyle habits, such as a healthy diet and physical activity, to reduce the incidence of dysglycaemia and associated cardiometabolic risk factors.

## Data Availability

The data sets used and/or analysed during the current study are available from the corresponding author on reasonable request.

## Abbreviations

T2DM: type 2 diabetes mellitus
WC: waist circumference
BMI: body mass index
SBP: systolic blood pressure
DBP: diastolic blood pressure
TC: total cholesterol
HDL-c: high density lipoprotein cholesterol
LDL-c: low density lipoprotein cholesterol
TG: triglycerides
WHR: ratio of waist circumference to hip circumference
Total/HDL-c ratio: ratio of TC to HDL-c levels
FBG: fasting blood glucose
PPG: postprandial glucose
IGT: impaired glucose tolerance
IFG: impaired fasting glucose
ROC: receiver operating characteristic
AIC: akaike information criterion
MI: myocardial infarction
